# Massive and massless Dirac fermions in Pb_1−x_Sn_x_Te topological crystalline insulator probed by magneto-optical absorption

**DOI:** 10.1038/srep20323

**Published:** 2016-02-04

**Authors:** B.A. Assaf, T. Phuphachong, V.V. Volobuev, A. Inhofer, G. Bauer, G. Springholz, L.A. de Vaulchier, Y. Guldner

**Affiliations:** 1 Département de Physique, Ecole Normale Supérieure, CNRS, PSL Research University, 24 rue Lhomond, 75005 Paris, France; 2 Laboratoire Pierre Aigrain, Ecole Normale Supérieure, CNRS, PSL Research University, Université Pierre et Marie Curie, Université Denis Diderot, 24 rue Lhomond, 75005 Paris, France; 3 Institut für Halbleiter und Festkörperphysik, Johannes Kepler Universität, Altenberger Strasse 69, 4040 Linz, Austria; 4 National Technical University, Kharkiv Polytechnic Institute, Frunze Ste. 21, 61002 Kharkiv, Ukraine

## Abstract

Dirac fermions in condensed matter physics hold great promise for novel fundamental physics, quantum devices and data storage applications. IV-VI semiconductors, in the inverted regime, have been recently shown to exhibit massless topological surface Dirac fermions protected by crystalline symmetry, as well as massive bulk Dirac fermions. Under a strong magnetic field (B), both surface and bulk states are quantized into Landau levels that disperse as B^1/2^, and are thus difficult to distinguish. In this work, magneto-optical absorption is used to probe the Landau levels of high mobility Bi-doped Pb_0.54_Sn_0.46_Te topological crystalline insulator (111)-oriented films. The high mobility achieved in these thin film structures allows us to probe and distinguish the Landau levels of both surface and bulk Dirac fermions and extract valuable quantitative information about their physical properties. This work paves the way for future magnetooptical and electronic transport experiments aimed at manipulating the band topology of such materials.

Topological crystalline insulators (TCI) are a novel class of materials that host an even number of Dirac surface states at points that are mirror symmetric with respect to a certain crystallographic plane^1^. The (111) and (100) surfaces of rocksalt IV-VI semiconductors Pb_1-x_Sn_x_Te and Pb_1-x_Sn_x_Se (Fig. 1(a)) were shown to possess such Dirac surface states^2^,^3^,^4^,^5^,^6^,^7^,^8^,^9^,^10^. In Pb_1-x_Sn_x_Te and Pb_1-x_Sn_x_Se, a band inversion occurs at the L-points of the Brillouin zone, as the Sn content is increased^11^,^12^,^13^. Since the L-points are mirror symmetric with respect to the (110) diagonal planes, such a band inversion results in the emergence of topological surface states (TSS) at 4 different points on the (100)^3^,^4^,^5^ and the (111) surfaces^7^,^8^,^9^,^14^,^15^. IV-VI semiconductors are thus 4-fold degenerate TCI where topology is governed by the symmetry of the crystal. As such, TCI hold a great potential for tunable Dirac electronics^16^,^17^,^18^,^19^, stemming from the inherent sensitivity of band topology on the crystal structure^2^,^20^, as well as from the highly mobile character of Dirac Fermions^21^,^22^. A complete understanding of the behavior of surface Dirac fermions in such materials and the ability to reliably distinguish them from the bulk carriers is, however, a necessary prerequisite to their development and implementation in potential devices^16^,^23^,^24^.

As in most other topological materials, the bulk states may also contribute in magnetotransport experiments as well as in optics. As shown in Fig. 1(b), the bulk carriers in (111) oriented Pb_1-x_Sn_x_Te populate 4 ellipsoidal carrier pockets, one of which is a longitudinal pocket whose major axis is parallel to the [111] direction, while the other three have their major axes tilted by ±70.5° with respect to the [111] direction. In total, one has to deal with a complex Fermi surface that comprises a longitudinal bulk ellipsoidal valley, three tilted oblique bulk valleys, a 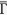-point surface Dirac valley, and three 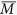-point surface Dirac valleys. The bulk states are also expected to be Dirac-like^25^, and may result in carrier mobilities that could be as high as what is expected for the topological surface states. Hence, in order to reliably identify the topological surface states and study them with conventional transport and optical probes, one needs to be able to understand and rule out contributions from bulk states that may contribute similar, if not identical signatures. Additionally, previous magnetooptical^26^,^27^ and transport studies^25^,^28^,^29^ have proven difficult the task of probing, identifying and assigning the Landau levels of topological materials. This is mainly due to the fact that the Fermi level in such systems is pinned to the bulk states, and the mobility is limited, hence requiring fields exceeding 15T to achieve clear Landau quantization^30^.

In this work, we performed detailed magneto-optical absorption measurements to map out the Landau level (LL) spectrum of the bulk and surface bands of high mobility (111) epitaxial Pb_1-x_Sn_x_Te (x = 0.45–0.47) films grown by molecular beam epitaxy (MBE). By lightly doping Pb_1-x_Sn_x_Te with Bi (about 10^19^ cm^−3^)^31^, we are able to compensate residual defect doping that results from (Pb, Sn) vacancies, and achieve a low carrier density without compromising the mobility. We can, hence, obtain bulk carrier densities that are close to 1 × 10^18^ cm^−3^ and mobilities of 10000 cm^2^/Vs. This results in a Landau quantization at relatively low magnetic fields (1.5T) and allows us to reliably map the LL spectrum of both bulk and surface states.

Magneto-optical measurements reveal a number of strong interband Landau level transitions that are well described by a massive Dirac dispersion model^25^. These transitions are associated with two types of bulk ellipsoidal Fermi surfaces – a longitudinal carrier valley and three-fold degenerate oblique valleys shown in Fig. 1(b). After reliably mapping out all bulk contributions, we are able to identify a cyclotron resonance feature pertaining to a massless Dirac state having a Fermi velocity v_f_ = 7.3 × 10^5^ m/s, attributed to the 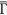-point Dirac cone. This is reproduced in two samples having slightly different carrier densities. Our results are in agreement with previous studies on the bulk bands in SnTe^32^, Pb_1-x_Sn_x_Te^33^, and PbTe^34^,^35^,^36^ and recent calculations of the band structure of the (111) surface states in TCI systems^9^,^14^.

## Results

### Growth and characterization

**Figure 1:**
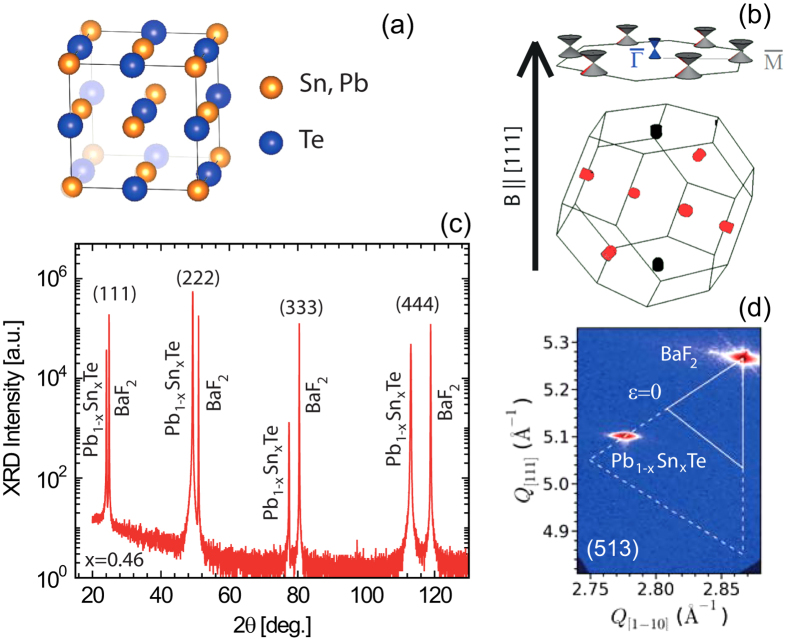
The structural properties of Pb_1-x_Sn_x_Te (111) films and sketch of their Brillouin zone. (**a**) Rocksalt (Fm-3m) crystal structure of Pb_1-x_Sn_x_Te highlighting a (111) Bragg plane in grey. (**b**) Sketch of the first Brillouin zone of Pb_1-x_ Sn_x_Te rotated so that a (111) plane points towards the top. The longitudinal bulk valleys are shown in black, and the oblique bulk valleys are shown in red. A sketch of the (111) topological surface Dirac cones is shown on top. The 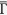 Dirac cone shown in blue has a larger Fermi velocity than the three 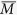 Dirac cones. (**c**) Symmetric x-ray diffraction scan along [111] (using Cu-Kα_1_ radiation) of the Pb_1-x_Sn_x_Te sample S1 with x = 0.46 showing the epitaxial structure of the layer with respect to the substrate. (**d**) Reciprocal space map taken around the asymmetric (513) Bragg reflection, evidencing that the epilayer is strain free, i.e. fully relaxed.

High mobility Pb_1-x_Sn_x_Te films are grown by molecular beam epitaxy (MBE) on cleaved (111) BaF_2_ substrates, in a Varian Gen II system with a base pressure of 10^−10^ mbar. Compound PbTe and SnTe effusion cells are employed to control the composition of the layer, through the PbTe-to-SnTe flux ratio that is determined using a quartz microbalance moved into the substrate position. For the present investigations, the Sn composition of the layers was fixed to x_Sn_ = 0.46 ± 0.01, which is well in the non-trivial TCI regime. The thickness was fixed at 2μm. Bismuth n-type doping was employed to compensate the intrinsic p-type carrier concentration (typically p > 10^19^ cm^−3^) arising in Pb_1-x_Sn_x_Te from native Sn and Pb vacancies. Bi was supplied from a compound Bi_2_Te_3_ effusion cell. When substitutionally incorporated on group IV lattice sites, Bi acts as singly charged donor and thus, can compensate the p-type background concentration. From a sample series with varying Bi doping levels, selected samples with carrier concentration around 1 × 10^18^ cm^−3^ and carrier mobilities of 10000 cm^2^/Vs are chosen for further investigation. Apart from the electrical measurements, all samples are characterized by high resolution X-ray diffraction (XRD) shown in Fig. 1(c,d) and atomic force microscopy (AFM, see supplement). From XRD (Fig. 1(c)), a clear Pb_1-x_Sn_x_Te {111} Bragg series can be observed, evidencing a perfect epitaxial relationship between layer and substrate. The layers are fully relaxed with practically zero residual strain as shown by the reciprocal space map around the asymmetric (513) Bragg reflection (Fig. 1(d)), where the Pb_1-x_Sn_x_Te peak is found to be located exactly on the zero strain line (ε = 0). The composition of the layers derived from XRD using the Vegard’s law agrees within ±1% with the nominal value.

### Magnetooptics-Bulk States

**Figure 2:**
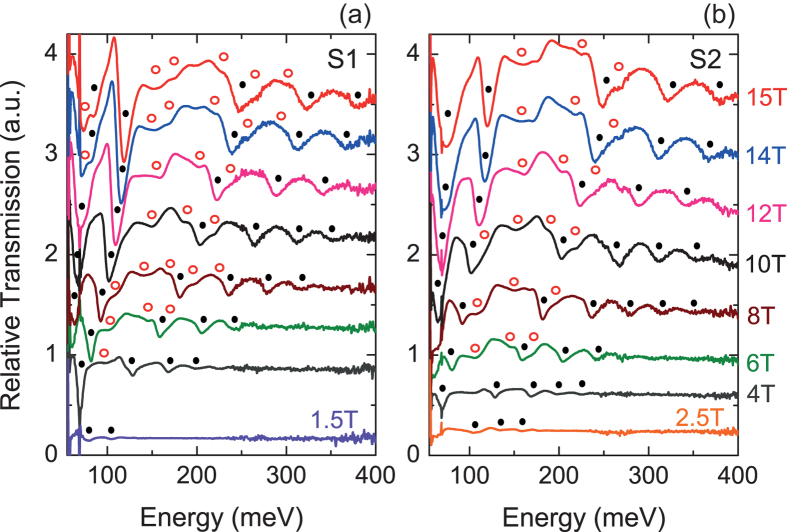
Magnetooptical spectra of Pb_0.54_Sn_0.46_Te (111) films. (**a**,**b**) Magneto-optical transmission spectra (calculated by dividing the raw spectra by the spectrum at B = 0T) as a function of energy, plotted for magnetic fields between 1.5T and 15T in sample S1 and 2.5T and 15T in S2. The full black circles and empty red circles respectively denote absorption minima corresponding to the longitudinal and oblique valleys. The curves are shifted vertically for clarity purposes. The slope in the baseline of the spectra originates from the response of the detector to the applied magnetic field. Its impact on the position of the minima is negligible, since it is smaller than our reported error bars.

Infrared magneto-optical spectroscopy experiments are performed on large pieces of two samples (S1 and S2), at 4.5K and up to B = 15T. The samples are exposed to radiation from a mercury lamp in the Faraday geometry, with the magnetic field parallel to the [111] direction. The transmitted signal is then collected using a Si composite bolometer and analyzed by a Fourier transform spectrometer. Figure 2(a,b) show typical infrared transmission spectra taken at different magnetic fields between 55 and 400 meV in both samples. A number of strong absorption minima that disperse with increasing magnetic field can be clearly seen. The strongest series marked by black dots is associated with the bulk longitudinal valley. Several other transitions marked by the red circles, can also be resolved and are attributed to bulk oblique valleys. Note that the mere fact that a strong and clear modulation is seen versus energy at fields as low as 1.5T is unambiguous evidence of the high mobility and low carrier density of the films.

**Figure 3:**
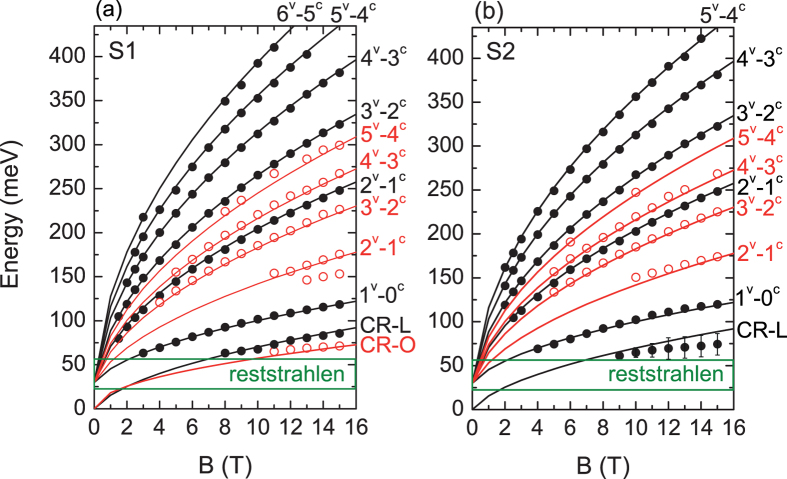
Experimental and theoretical Landau fan diagrams of Pb_0.54_Sn_0.46_Te (111) films. Experimental Landau fan diagram for the longitudinal (full black circles) and oblique (empty red circles) bulk valleys for S1 (**a**) and S2 (**b**). Solid lines are theoretical curves obtained from a massive Dirac model (Eqs. (2, 3)) with v_f_  = 7.3 × 10^5^ m/s for the longitudinal valley (black lines) and v_f_ = 5 × 10^5^m/s for the oblique valleys (red lines) and ∆ ≈ 15 meV. The BaF_2_ substrate reststrahlen band (22 to 55 meV) is shown in green. CR denotes the cyclotron resonance absorption. Interband absorptions N^v^–(N-1)^c^ are denoted by their respective Landau index N and their band index (c for the conduction band and v for valence band). The CR-data points in S1 are extracted using a multi-peak fitting method described later in the text. CR-L and CR-O respectively denote the CR originating from the longitudinal and the oblique valleys.

Figure 3(a,b) respectively show the detailed analysis performed for S1 and S2, whereby absorption energy minima are identified (Fig. 2(a,b)) and then plotted as a function of magnetic field in order to construct a Landau fan diagram. A massive Dirac model is then used to fit the data for both types of valleys^25^,^37^,^38^. The LL energies at 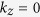 (B and **z**||[111]) are sufficient to describe the magnetooptical absorption spectra since the joint density of states is optimal for 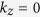. Taking the zero energy at the midgap, the LL energies given by the massive Dirac model are:


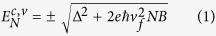


The ± sign refers to the conduction 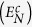 and valence 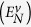 band levels respectively and ∆ is equal to half of the band gap E_g_, 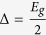. The mass of the Dirac Fermions is then given by 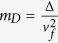, where v_f_ is the Fermi velocity. *N* denotes the Landau index, *e* is the fundamental electronic charge, and *ħ* is the reduced Planck constant. The absorption energies for interband transitions are then given by:


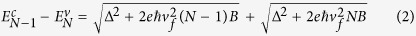


The cyclotron resonance (CR) as a function of field satisfies the intraband equivalent:


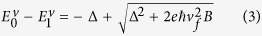


Note that a similar expression can also be derived from a two-band ***k.p*** model that includes spin degeneracy^36^,^37^,^39^,^40^,^41^. For the Sn content that we considered in this work (x ≈ 0.46)^42^, a two-band approach is justified, since the conduction (*L*^*6+*^) and valence (*L*^*6−*^) bands are mirror images of each other and the far-band contributions^13^,^38^ can be neglected for an energy gap of 30meV. In this case, the LL spin splitting is known to be almost equal to the cyclotron energy, so that the N^th^ LL of one spin component coincides with the (N+1)^th^ LL of the other^13^,^36^,^43^. This also follows directly from the Dirac Hamiltonian^37^ and is even well known to be valid in general for Dirac electron propagating in vacuum. The spin degeneracy will therefore not give additional transmission minima as discussed in more detail in the supplement.

The black lines in Fig. 3 are the calculated magneto-optical transition energies for the longitudinal valley using the massive Dirac model described earlier. A very good agreement between the theory and the experimental interband data is obtained for v_f_ = (7.3 ± 0.1) × 10^5^ m/s and ∆ = 15 ± 3 meV for both samples. The energy gap E_g_ = 2∆ ≈ 30 ± 6 meV agrees with the value expected for Pb_0.54_Sn_0.46_Te. The Fermi velocity is also, in excellent agreement with the **k.p** matrix element determined in ref. 13. (see supplement). The band edge mass is found to be equal to 0.005 m_0_. The longitudinal inter-LL transitions 1^v^–0^c^ and 2^v^–1^c^, where v and c respectively denote the valence and conduction band level, are measured down to B≈4T and 2T respectively, indicating a Fermi energy of about E_F_≈40 ± 5 meV below the valence band edge of the longitudinal valley.

The additional minima shown as open red circles in Fig. 2(a,b) are associated with carriers in the oblique valleys. As mentioned before, the Fermi surface of (111) oriented Pb_1-x_Sn_x_Te is highly anisotropic^34^,^35^,^36^. The oblique valleys result in an anisotropy factor K = 10, defined as the square of the ratio of the maximum and minimum cross-sectional areas of the 3D Fermi surface (Fig. 1(b)). This agrees with previous studies on the (Pb, Sn) chalcogenides^32^,^34^,^35^,^36^.

The red lines in Fig. 3 are the calculated magneto-optical absorption energies for the oblique valleys using the massive Dirac model. A good agreement is found between the data and the model, for a Fermi velocity v’_f_ = 5 × 10^5^ m/s for both samples. This corresponds to a ratio of 1.46 between the Fermi velocity of the longitudinal and the oblique valleys, in agreement with the expected Fermi surface anisotropy (see supplement). For ∆ = 15meV this yields a mass equal to 0.011 m_0_. The transition 3^v^–2^c^ is measured down to B = 4T, indicating a Fermi energy E_F_ ≈ 50 ± 5meV below the valence band edge of the oblique valley for both S1 and S2. Note that it is not surprising that the Fermi level be slightly different in different valleys, as the Pb_1-x_Sn_x_Te/BaF_2_(111) system is known to exhibit a strong thermal expansion mismatch at low temperature, that may shift the oblique bands up in energy with respect to the longitudinal bands. This has been observed in IV–VI epilayers and quantum wells grown on BaF_2_(111)^44^,^45^,^46^.

Note that the CR splitting observed in S1 above 11T in Fig. 2(a), is due to the simultaneous presence of both the longitudinal and the oblique CR lines. The critical magnetic field, above which the (1^v^–0^v^) oblique CR line is observed, is in agreement with the energy at which the N = 1 LL crosses the Fermi level. We are, however, unable to resolve both CR lines in S2. The average peak position is thus reported with a large error bar that takes into account the broadening of the peak as a result of the presence of two transitions at those energies.

### Magnetooptics – Surface states

**Figure 4:**
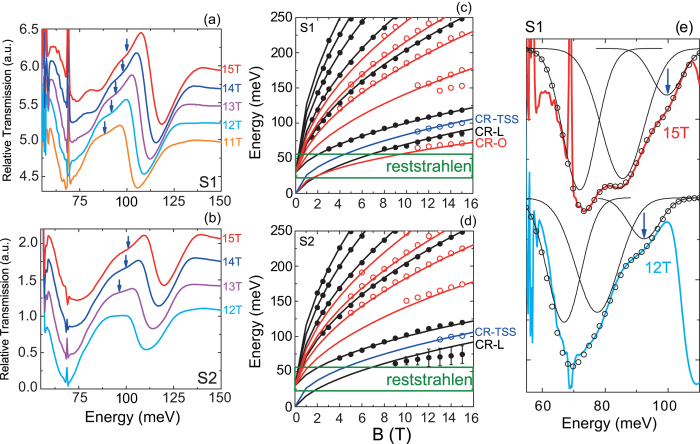
The cyclotron resonance of the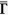 point topological surface state. Absorption minima at low energy for both S1 (**a**) and S2 (**b**) showing the cyclotron resonance feature attributed to the topological surface states (CR-TSS) between the stronger bulk absorption minima. Landau fan diagrams for S1 (**c**) and S2 (**d**) including the feature interpreted as the CR-TSS in blue. Solid blue lines are obtained by theoretically calculating the CR transition of massless Dirac Fermions having v_f_ = 7.3 × 10^5^m/s. The error bars given in (**d**) take into account the broadening of the bulk CR minimum seen in (**b**). (**e**) Multi-peak fitting in S1 for spectra taken at 15T (red data) and 12T (blue data). Three Gaussian curves resulting from the fitting procedure are shown. The resultant sum of the three peaks (empty black circles) gives an excellent fit to the data at both fields. The blue arrow refers to the peak attributed to the CR-TSS transition. The bulk-CR data points in S1 are also extracted using this method.

We now turn our attention to a CR feature that seems to evade the expected physics of the bulk longitudinal and oblique valleys. Figure 4(a,b) present a zoomed in view of the spectra at high magnetic fields (11–15T) in the range 55–150 meV. Besides the main absorptions associated with the CR and the first interband transition of the longitudinal bulk valley, an absorption indicated by the blue arrows is measured.

This absorption (blue points in Fig. 4(c,d)) is interpreted as the CR of the topological surface states (CR-TSS) at the 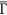 point. The blue lines are the calculated transition energies for a massless Dirac model using the same Fermi velocity as the bulk longitudinal valley, v_f_ = 7.3 × 10^5^ m/s, 
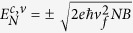
. This gives:


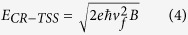


Good agreement is again found between the model and the experimental data for the CR-TSS feature as shown by the blue line in Fig. 4(c,d). Moreover, the fact that we do not observe any CR-TSS transitions below 10T in S1 (12T in S2) agrees with the estimated Fermi levels for bulk bands (40 ± 5 meV below the band edge), as accordingly the TSS Fermi level would be close to 60 meV below the Dirac point. This fact actually reinforces our interpretation. Note, however, that the TSS interband transitions cannot be resolved in our experiments because they are located at the same energy as the intense interband transitions of the longitudinal bulk valley. Indeed, the LL energies of the TSS 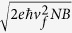 coincide with those of the massive bulk fermions 
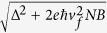
 when 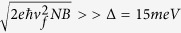. This is the case in our measurements for N ≥ 1.

Due to the apparent low intensity of the CR-TSS in the raw signal, we have used a multi-peak fitting scheme to identify the position of the transition minima in each case. This procedure is shown in Fig. 4(e). We can clearly identify three peak features; two strong features resulting from the CR of the bulk states as well as a smaller one indicated by the blue arrow that we attribute to the CR-TSS. The feature marked by the blue arrow is essential to explain the dip observed in the raw signal, and is sufficiently large in intensity to be reliably accounted for in the analysis. At 15T we find the CR-TSS at 100 ± 2 meV, whereas at 12T it is located at 92 ± 2 meV. The blue arrows and the CR data points in Fig. 3 and 4 are identified and placed using this method.

## Discussion

**Figure 5:**
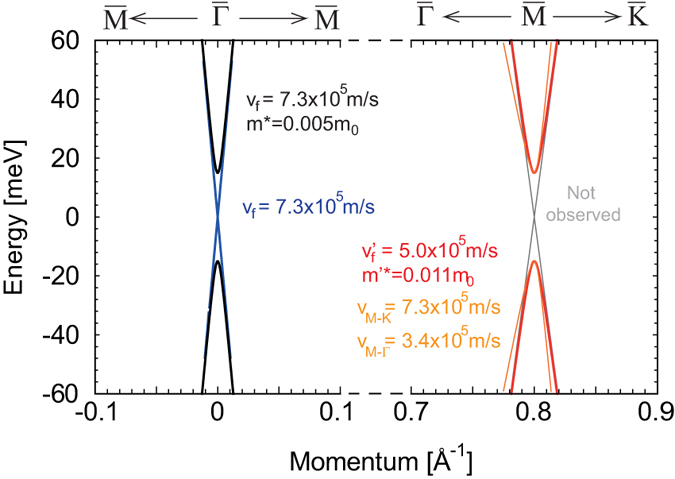
Summary of band parameters extracted in this study. Band structure plotted using the parameters extracted for the bulk massive Dirac fermions (black, red and orange) and the topological massless Dirac fermions (blue).

In sum, we are able to map out the band structure of Bi-doped Pb_0.54_Sn_0.46_Te in the vicinity of the band gap. Our interpretation is summarized in Fig. 5. The longitudinal bulk valley is found to satisfy a massive Dirac Hamiltonian with a Fermi velocity v_f_ = (7.3 ± 0.1) × 10^5^ m/s and an effective mass equal to m* = 0.005 m_0_. This Fermi velocity is in excellent agreement with the **k.p** matrix element determined in ref. 13. (see supplement). The bulk oblique valleys satisfy that same massive Dirac Hamiltonian with v’_f_ = (5.0 ± 0.1) × 10^5^ m/s and an effective mass m’* = 0.011m_0_. Finally, a CR that satisfies a massless Dirac model with v_f_ = (7.3 ± 0.1) × 10^5^ m/s is observed and attributed to the TSS 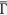-point Dirac cone. We did not see any signal from LL pertaining to the 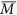-point Dirac cones. The reason behind this might be the fact that the Fermi velocity for 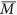-point Dirac cones coincides with that of the oblique bulk valleys. In that case, the interband transitions of the 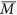-point Dirac cone will overlap with those of the oblique bulk valleys. The CR signal for 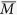-point Dirac cones is likely to occur at energies similar to where the bulk-CR lines were observed. It is also expected to be weaker in intensity; we are thus not able to resolve it.

Finally, one has to keep in mind that the Fermi velocity of the oblique valleys is expected to be anisotropic^9^. This is due to the fact that the oblique valleys are tilted by 70.5° with respect to the [111] direction and the applied magnetic field. Our experimental value of v’_f_ = (5.0 ± 0.1) × 10^5^ m/s is thus an effective result given by 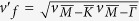. Here 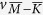
denotes the Fermi velocity along the 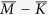 direction, expected to be almost equal to that measured at the 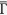-point – (7.3 ± 0.1) × 10^5^ m/s^9^. One can thus extract 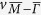, the Fermi velocity along the 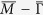 direction. We get 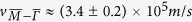. The details of this geometric argument are discussed in the supplement.

In conclusion, we have mapped out the Landau level spectrum of high mobility Bi-doped Pb_0.54_Sn_0.46_Te (111) epilayers grown on BaF_2_. The high mobility and low carrier density achieved in these films lead to a Landau quantization at about 1.5T. This allows us to reliably map out the LL at low energies. The bulk longitudinal and oblique valleys of Pb_0.54_Sn_0.46_Te can be reliably interpreted by a massive Dirac fermion model. The cyclotron resonance of the topological surface 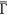-point Dirac cone is also revealed above 10T. Its dispersion is characteristic of massless Dirac fermions having a Fermi velocity v_f_ = 7.3 × 10^5^ m/s. The interband transitions corresponding to the TSS cannot be resolved as they overlap those of the bulk longitudinal valley. Our results provide vital information about the effective masses and Fermi velocities of different bands in Pb_0.54_Sn_0.46_Te, most notably the 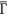-Dirac point, and will be of great use to future transport experiments studying quantum oscillations and coherent transport.

## Methods

Bi-doped Pb_1-x_Sn_x_Te films were grown by molecular beam epitaxy in a Varian Gen II MBE system on cleaved BaF_2_(111) substrates. High purity PbTe, SnTe and Bi_2_Te_3_ source materials were used. High resolution X-ray diffraction measurements were then performed using Cu-Kα_1_ radiation in a Seifert XRD3003 diffractometer, equipped with a parabolic mirror, a Ge(220) primary beam Bartels monochromator and a Meteor 1D linear pixel detector. Preliminary transport measurements were performed at 77K in order to determine the carrier density and mobility. Further transport characterization was performed at 2K and up to 8T in an Oxford Instruments 1.5K/9T cryostat. Magneto-optical absorption experiments were performed in an Oxford Instruments 1.5K/15T cryostat at 4.2K. Spectra were acquired using a Bruker Fourier transform spectrometer.

## Additional Information

**How to cite this article**: Assaf, B.A. *et al.* Massive and massless Dirac fermions in Pb_1-x_Sn_x_Te topological crystalline insulator probed by magneto-optical absorption. *Sci. Rep.* **6**, 20323; doi: 10.1038/srep20323 (2016).

## Supplementary Material

Supplementary Information
