# Supplementary material for: Massive and massless Dirac fermions in Pb1−xSnxTe topological crystalline insulator probed by magneto-optical absorption
Source: Sci Rep. 2016 Feb 4;6:20323. doi: 10.1038/srep20323 (PMC4740886; doi:10.1038/srep20323)
Supplement: Supplementary Information [file srep20323-s1.pdf]

Supplementary material for Massive and Massless Dirac fermions in $Pb_{1-x}Sn_xTe$ topological crystalline insulator probed by magneto-optical absorption

B.A. Assaf, T. Phuphachong, V.V. Volobuev, A. Inhofer, G. Bauer, G. Springholz, L.A. de Vaulchier, Y. Guldner

1. Transport parameters and structural characterization

The Hall carrier density and mobility were measured in both samples at 2K. Hole-type carriers were found to contribute for both. The carrier densities deduced from a high-field Hall measurement (up to 8T) were measured to be slightly different in the two samples: $p=1.0\times10^{18}cm^{-3}$ for S1 and $p=1.2\times10^{18}cm^{-3}$ for S2. The Hall mobility was found to be equal to 4800 cm^2/Vs in S1 and 10500 cm^2/Vs in S2. Let us keep in mind, however, that this is an effective mobility that is averaged over all band and valley contributions. The carrier mobility in the longitudinal bulk valley which depends only on the transverse effective mass and in the Dirac cones may thus be larger than these reported values.

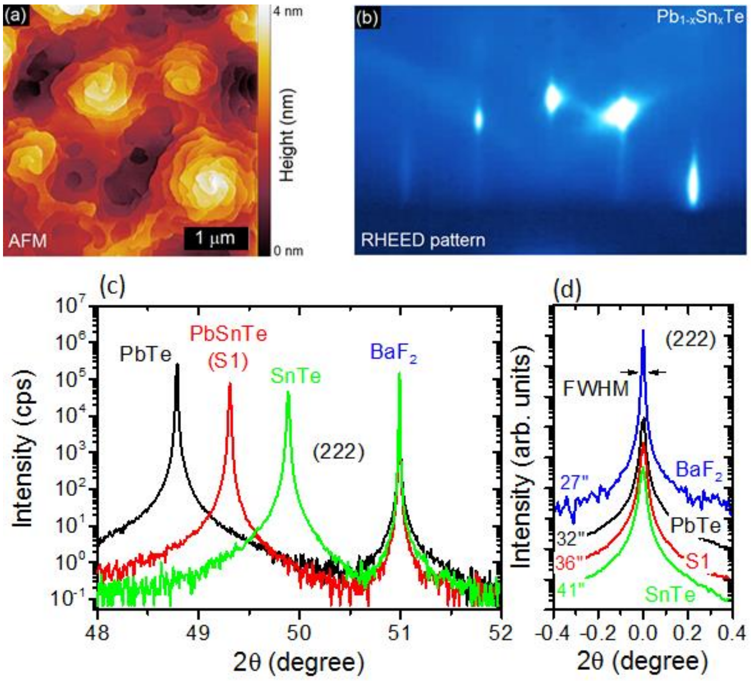

Supplementary Figure 1. (a) Atomic force microscopy (AFM) image of a 2µm $Pb_{1-x}Sn_xTe$ epilayer on BaF_2(111) substrate. (b) Reflection high energy electron diffraction pattern obtained in-situ during the growth of a $Pb_{1-x}Sn_xTe$ layer on a BaF_2 (111) substrate, showing a series of equally spaced streaks, evidence of a high quality (111) oriented epitaxial layer. (c) High resolution x-ray diffraction pattern recorded in 4-crystal geometry on the $Pb_{0.54}Sn_{0.46}Te$ epilayer S1 and pure PbTe and SnTe epilayers grown under the same conditions on BaF_2 (111) substrates. From the peak position, the lattice constants and thus, the chemical composition is derived using Vegard's law. (d) Comparison of the FWHM of the (222) diffraction peak in all three samples. The practically identical FWHM (shown on the left side of the curves) found in the three samples proves the absence of any compositional alloy fluctuations in the ternary $Pb_{0.54}Sn_{0.46}Te$ layer.

The samples are additionally characterized by AFM as shown in Suppl. Fig. 1(a). A number of screw-type dislocations can be seen in the AFM image. The surfaces, however, exhibit only single monolayer steps around growth spirals induced by the dislocations, originating from the lattice mismatch with the BaF_2 substrates ($\Delta a/a$ = 3.07 %). Locally, terraces that are several hundreds of nanometers in length and width are seen. Finally, a RHEED pattern of the $Pb_{1-x}Sn_xTe$ layer is shown in Suppl. Fig. 1 (b). A series of streaks is seen in the figure evidencing the high crystalline quality of the film. Additionally, a through XRD comparative study is performed on three samples, S1, a PbTe epilayer and a SnTe epilayer, all grown under identical conditions on BaF_2 (111). The (222) Bragg peak for the three samples is shown in Suppl. Fig. 1(c). The peak position changes upon Sn doping of PbTe due to the changing lattice constant, as dictated by Vegard's law. We then compared the FWHM of the (222) Bragg peaks in Suppl. Fig 1(d). It is evidently clear that the FWHM (36'') of the (222) peak for sample S1 is identical to that of SnTe (41'') and PbTe (32''), even in the presence of Bi-doping. We can thus rule out the existence of compositional inhomogeneities and concentration gradients in the sample, therefore confirming that a single phase is successfully formed in S1.

2. Equivalence between the two-band k.p model and the massive Dirac model in $Pb_{1-x}Sn_xTe$

The **k.p** and Dirac Hamiltonians in IV-VI semiconductors

According to previous studies [1,2,3,4,5,6,7] a six-band **k.p** Hamiltonian provides a good description of the electronic band structure of PbTe, and consequently other IV-VI semiconductors that crystallize in the rocksalt structure with direct energy gaps at the L-points of the Brillouin zone. For narrow (nearly zero) gap alloys of SnTe and PbTe such as $Pb_{0.54}Sn_{0.46}Te$, it can, however, be argued that a two-band description is sufficiently accurate. A two-band **k.p** Hamiltonian matrix is given by:

$$\begin{pmatrix} \frac{E_g}{2}-E & 0 & \frac{\hbar}{m_0}P_{\parallel}k_z & \frac{\hbar}{m_0}P_{\perp}k_- \\ 0 & \frac{E_g}{2}-E & \frac{\hbar}{m_0}P_{\perp}k_+ & -\frac{\hbar}{m_0}P_{\parallel}k_z \\ \frac{\hbar}{m_0}P_{\parallel}k_z & \frac{\hbar}{m_0}P_{\perp}k_- & -\frac{E_g}{2}-E & 0 \\ \frac{\hbar}{m_0}P_{\perp}k_+ & -\frac{\hbar}{m_0}P_{\parallel}k_z & 0 & -\frac{E_g}{2}-E \end{pmatrix} \begin{pmatrix} f_1 \\ f_2 \\ f_3 \\ f_4 \end{pmatrix} = 0 \quad (1)$$

Here E_g is the band gap, $P_{\parallel}$ and $P_{\perp}$ are respectively the longitudinal and transverse momentum matrix elements. (k_x, k_y, k_z) are the wavevector coordinates and $k_{\pm}=k_x \pm ik_y$. m_0 is the electron rest mass. We define the z-axis to be oriented along the [111] direction. The magnetic field is applied in the same direction and the magneto-optical transitions occur at k_z=0. We thus get:

$$\begin{pmatrix} \frac{E_g}{2}-E & 0 & 0 & \frac{\hbar}{m_0}P_{\perp}k_- \\ 0 & \frac{E_g}{2}-E & \frac{\hbar}{m_0}P_{\perp}k_+ & 0 \\ 0 & \frac{\hbar}{m_0}P_{\perp}k_- & -\frac{E_g}{2}-E & 0 \\ \frac{\hbar}{m_0}P_{\perp}k_+ & 0 & 0 & -\frac{E_g}{2}-E \end{pmatrix} \begin{pmatrix} f_1 \\ f_2 \\ f_3 \\ f_4 \end{pmatrix} = 0 \quad (2)$$

This is equivalent to solving the two following decoupled matrices:

$$\begin{pmatrix} E_g/2-E & \frac{\hbar}{m_0}P_\perp k_- \\ \frac{\hbar}{m_0}P_\perp k_+ & -E_g/2-E \end{pmatrix}\begin{pmatrix} f_1 \\ f_4 \end{pmatrix}=0 \quad \text{and} \quad \begin{pmatrix} E_g/2-E & \frac{\hbar}{m_0}P_\perp k_+ \\ \frac{\hbar}{m_0}P_\perp k_- & -E_g/2-E \end{pmatrix}\begin{pmatrix} f_2 \\ f_3 \end{pmatrix}=0 \qquad (3)$$

Recalling that $k_\pm=k_x\pm ik_y$, the two matrices can be rewritten in the form of a massive Dirac Hamiltonian matrix where the Fermi velocity is defined as $v_f=\frac{P_\perp}{m_0}$:

$$\begin{pmatrix} E_g/2-E & \hbar v_f(k_x-ik_y) \\ \hbar v_f(k_x+ik_y) & -E_g/2-E \end{pmatrix}\begin{pmatrix} f_1 \\ f_4 \end{pmatrix}=0 \qquad (4)$$

We can thus calculate v_f from the value of $\frac{P_\perp^2}{m_0}=2.975eV$ given by Bauer in ref. 5:

$$v_f=\sqrt{\frac{2.975\times1.6\times10^{-19}}{9.1x10^{-31}}}=7.23\times10^5 m/s \qquad (5)$$

We measured v_f = (7.3±0.1)x10^5m/s for the longitudinal valley, in excellent agreement with the calculated value.

Landau levels in the two-band **k.p** model and the massive Dirac model in $Pb_{1-x}Sn_xTe$

When a magnetic field is applied, $\vec{k}$ is replaced by $\vec{k}-e\vec{A}/\hbar$. According to ref. 5 Landau quantization in the two-band **k.p** model results in the following:

$$\begin{pmatrix} E_g/2-E & \sqrt{E_g\hbar\omega\left(n+\frac{1}{2}\right)+E_g\frac{g\mu_B B}{2}} \\ \sqrt{E_g\hbar\omega\left(n+\frac{1}{2}\right)+E_g\frac{g\mu_B B}{2}} & -E_g/2-E \end{pmatrix}\begin{pmatrix} \varphi_1 \\ \varphi_2 \end{pmatrix}=0 \qquad (6)$$

Here ω is the cyclotron frequency, *g* is the g-factor and μ_B the Bohr magneton. φ_1 and φ_2 are the harmonic oscillator wavefunctions (φ_n and φ_{n+1}). It can also be shown that for Dirac electrons as well as in the two-band **k.p** approximation the spin splitting is equal to the cyclotron energy,[4,5,7] which gives:

$$\begin{pmatrix} E_g/2-E & \sqrt{E_g\hbar\omega(n+1)} \\ \sqrt{E_g\hbar\omega(n+1)} & -E_g/2-E \end{pmatrix}\begin{pmatrix} \varphi_1 \\ \varphi_2 \end{pmatrix}=0 \qquad (7)$$

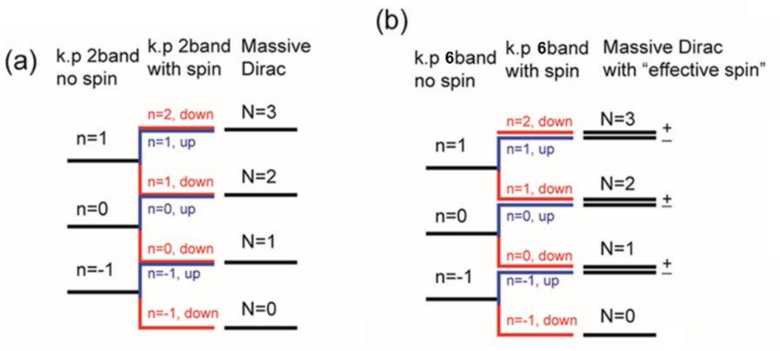

Supplementary Figure 2. Illustration of the Landau level spectrum obtained from (a) a two-band **k.p** model (and its equivalent, the Massive Dirac model) and (b) a six-band **k.p** model (and its equivalent, the Massive Dirac model that includes an "effective spin").

If we define $\Delta \equiv E_g/2$, and $v_f^2 = \Delta/m^*$, where m* is the effective mass in $\omega = eB/m^*$, and redefine our Landau index n+1 to be equal to N, we further simplify the notation to yield:

$$\begin{pmatrix} \Delta - E & \sqrt{2\hbar v_f^2 eBN} \\ \sqrt{2\hbar v_f^2 eBN} & -\Delta - E \end{pmatrix} \begin{pmatrix} \varphi_1 \\ \varphi_2 \end{pmatrix} = 0 \qquad (8)$$

This is the Landau level matrix of a massive Dirac fermion band dispersion of gap equal to 2Δ. We recover the massless equivalent by setting Δ=0.

The LL energies are then given by:

$$E_N = \pm\sqrt{\Delta^2 + \left(2\hbar v_f^2 eBN\right)} \qquad (9)$$

Note that in both the two-band **k.p** model and the massive Dirac model, all LL are spin degenerate except the N=0 level which is non-dispersive and is comprised of a single spin component as illustrated in Suppl. Fig. 2(a).[5,8]

Far band contributions are negligible in small gap compounds

In this entire study, we have neglected the contribution of the far bands. This is justified by the fact that the band gap in $Pb_{0.54}Sn_{0.46}Te$ is about 30meV and thus favors interactions between the highest valence band and lowest conduction band. The far-band correction to the two-band **k.p** model is discussed in detail in ref. 4 and ref. 5 for trivial IV-VI semiconductors. Small corrections to the cyclotron mass and g-factor result from far-band terms in the Hamiltonian. Such far band contributions have not, however, been determined for $Pb_{0.54}Sn_{0.46}Te$. We are thus not capable of providing a detailed quantitative analysis of their impact in our case.

If we use the parameters provided for PbTe in ref. 5 for the 6-band terms, the far-band corrections can be approximated. Corrections on the order of 5% are estimated for the effective masses. The g-factor corrections are even smaller and do not exceed 2%. They result in corrections in energy that are two orders of magnitude smaller than the energies measured in the case of the interband and intraband transitions at high magnetic field. The 6-band treatment will also lift the degeneracy of the N≥1 levels, and yield an "effective spin splitting" as shown in Suppl. Fig. 2(b). Both impacts of the 6-band model are negligible within our experimental resolution.

On a separate note, far-band contributions can yield a crossing of the conduction and valence N=0 levels at very high magnetic fields, for negative band gap (topologically non-trivial) alloys such as $Pb_{0.54}Sn_{0.46}Te$.

This has been measured in $Pb_{1-x}Sn_xSe$[9] and is briefly discussed by Bauer in ref. 5 (and references therein). The crossing is similar to what has been hinted to as the effect of a strong magnetic field on the bulk Landau levels of Bi_2Se_3 in the appendix of Bernevig and Hughes.[10] This, however, cannot be verified by our measurements.

3. Fermi surface geometry in $Pb_{1-x}Sn_xTe$

As shown in Fig. 1(b) and Suppl. Fig. 3, $Pb_{1-x}Sn_xTe$ alloys possess four carrier valleys, one of which – parallel to the [111] direction – is called longitudinal valley. The longitudinal valley is parallel to the growth axis and the direction of the applied magnetic field. The remaining three valleys – referred to as the oblique valleys are tilted by an angle of approximately 70.5° with respect to the [111] direction. When applying a magnetic field in the [111] direction, the Landau level spacing (and the Shubnikov-de-Haas frequency) of each valley is proportional to the area resulting from the cross-section of each respective valley and a (111) plane. For the longitudinal valley this results in a circle of diameter *2a* - the minor axis of the 3D Fermi ellipsoid. For any tilted valleys, that cross-section is an ellipse of minor axis equal to *2a* and major axis equal to *2k(θ)*, where θ=70.5° is the tilt angle of the oblique valleys when B is parallel to the [111] direction. The area anisotropy factor is $K=\left(\frac{b}{a}\right)^2=10$ for $Pb_{1-x}Sn_xTe$ when x=0.40[11] (*2b* is the major axis of the 3D ellipsoid). We find for any angle θ:

$$k(\theta)=a\sqrt{\frac{K}{(K-1)\cos^2(\theta)+1}} \qquad (10)$$

$$k(70.5^\circ)=2.2a$$

The resulting mass anisotropy between the oblique (m'*) and the longitudinal valleys (m*) is then m'* ≈ 2.2m*. The Fermi velocity anisotropy is then given by $v_f = 1.48v'_F$.

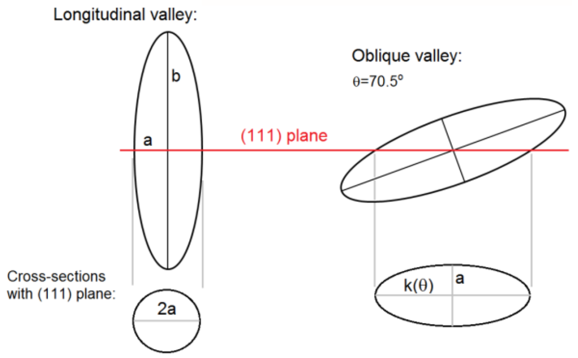

Supplementary Figure 3. Illustration of the Fermi surface geometry. The oblique valley is tilted by 70.5° with respect to the [111] axis and yields an elliptical cross-section with the (111) plane. The figure is not on scale.

References:

1. Dimmock, J. k.p theory for the conduction and valence bands of $Pb_{1-x}Sn_xTe$ and $Pb_{1-x}Sn_xSe$ alloys. in *Proceedings of the International Conference on the Physics of Semimetals and Narrow Gap Semiconductors, 1969, edited by D. L. Carter and R. T. Bate* 319 (Pergamon, New York, 1971).

2. Mitchell, D. L. & Wallis, R. F. Theoretical energy-band parameters for the lead salts. *Phys. Rev.* **151,** 581–595 (1966).

3. Grisar, R., Burkhard, H., Bauer, G. & Zawadzki, W. Magnetooptical transitions and band parameters of PbTe. in *Proceedings of the International Conference on the Physics of Narrow Gap Semiconductors, Qarsazo, edited by J. Bauluszkiewicz* 115 (PWN-Polish Scientific, Warsaw, 1978).

4. Burkhard, H., Bauer, G. & Zawadzki, W. Band-population effects and intraband magneto-optical properties of a many-valley semiconductor: PbTe. *Phys. Rev. B* **19,** 5149–5159 (1979).

5. Bauer, G. in *Narrow Gap Semiconductors Physics and Applications* 427–446 (1980). doi:10.1007/3-540-10261-2_56. *Ed. by W. Zawadzki*

6. Bauer, G., Pascher, H. & Zawadzki, W. Magneto-optical properties of semimagnetic lead chalcogenides. *Semicond. Sci. Technol.* **7,** 703–723 (1999).

7. Pascher, H., Bauer, G. & Grisar, R. Magnetooptical investigations and four-wave-mixing spectroscopy of PbSe. *Phys. Rev. B* **38,** 3383–3390 (1988).

8. Liang, T. *et al.* Evidence for massive bulk Dirac fermions in Pb(1−x)Sn(x)Se from Nernst and thermopower experiments. *Nat. Commun.* **4,** 3696 (2013).

9. Calawa, A., Dimmock, J., Harman, T. & Melngailis, I. Magnetic Field Dependence of Laser Emission in $Pb_{1-x}Sn_xSe$ Diodes. *Physical Review Letters* **23,** 7–10 (1969).

10. Bernevig, B. A. & Hughes, T. L. *Topological Insulator and Topological Superconductors*. (Princeton University Press, 2013).

11. Yusheng, H. & Graissie, A. D. C. Te alloys. III. Implications for the Fermi surface of SnTe. *Journal of Physics F: Metal Physics* **15,** 363–376 (2000).
